# Surgical treatment of patent ductus arteriosus in neonates

**DOI:** 10.1186/1749-8090-10-S1-A184

**Published:** 2015-12-16

**Authors:** Rui Almeida, Paula Futagami, Maria Silva

**Affiliations:** 1 Faculdade Assis Gurgacz, Cascavel, Paraná, Brazil; 2 Parana Western State University, Cascavel, Paraná, Brazil

## Background/Introduction

The patent ductus arteriosus is a congenital disorder characterized by incomplete closure or non-closure of the ductus arteriosus after birth. Surgical treatment, in the neonatal period, is indicated when the clinical treatment failed or there is contra-indication for it.

## Aims/Objectives

To identify the morbidity and mortality of neonates submitted to cardiac surgery for correction of patent ductus arteriosus, in a non pediatric unit of cardiovascular surgery.

## Method

A retrospective study of 17 neonatal patients, undergoing surgical correction of patent ductus arteriosus, was performed, in a period of eight years, from December 2007 to March 2015.

## Results

Female gender was predominant in 80% and the mean gestational age was 27.71 ± 2,95 weeks. The mean birth weight was 1324.32 ± 857,37 g, being the less weight 480g. The diameter of the ductus arteriosus was in average 2.82 mm (DP ± 0,94). Associated pathologies were present in 80% of the patients and 64.70% underwent premedical treatment. There was no operative mortality in this group of patients. Time of hospital stay in these patients was related to gain of weight, being two kilos the minimum to be discharged. Due to this the mean hospital stay after the correction of the heart defect was 97 days. Three patients died in ICU due to complications of other congenital malformations, such as neurological and gastrointestinal.

## Discussion/Conclusion

The surgery is the recommended treatment after failure of conventional medical treatment. It presents good results and no mortality, related to the cardiac surgery.

